# Effect of Vitamin D and Docosahexaenoic Acid Co-Supplementation on Vitamin D Status, Body Composition, and Metabolic Markers in Obese Children: A Randomized, Double Blind, Controlled Study

**DOI:** 10.3390/nu14071397

**Published:** 2022-03-27

**Authors:** Valentina De Cosmi, Alessandra Mazzocchi, Veronica D’Oria, Alessandro Re, Giulia Carla Immacolata Spolidoro, Gregorio P. Milani, Cristiana Berti, Silvia Scaglioni, Claudia Giavoli, Silvia Bergamaschi, Giulia Rodari, Eriselda Profka, Roberto Colombo, Carlo Agostoni

**Affiliations:** 1 Department of Clinical Sciences and Community Health, University of Milano, 20122 Milano, Italy; valentina.decosmi@unimi.it (V.D.C.); alessandra.mazzocchi@unimi.it (A.M.); giulia.spolidoro@unimi.it (G.C.I.S.); claudia.giavoli@unimi.it (C.G.); carlo.agostoni@unimi.it (C.A.); 2 Fondazione IRCCS Cà Granda Ospedale Maggiore Policlinico, Anestesia e Terapia Intensiva Donna-Bambino, 20122 Milano, Italy; veronica.doria@policlinico.mi.it; 3 Servizio di Alimentazione Pediatrica ASST Rhodense POT Bollate, 20024 Milano, Italy; re.alessandro.milano@gmail.com (A.R.); rrcolombo@asst-rhodense.it (R.C.); 4 Pediatric Unit, Fondazione IRCCS Cà Granda Ospedale Maggiore Policlinico, 20122 Milano, Italy; cristiana.berti@policlinico.mi.it; 5 Fondazione de Marchi Department of Pediatrics, Fondazione IRCCS Cà Granda Ospedale Maggiore Policlinico, 20122 Milano, Italy; silviascaglioni50@gmail.com; 6 Endocrinology Unit, Fondazione IRCCS Cà Granda Ospedale Maggiore Policlinico, 20122 Milano, Italy; bergamaschi.endocrinologia@gmail.com (S.B.); giulia.rodari@policlinico.mi.it (G.R.); eprofka@gmail.com (E.P.); 7 Pediatric Intermediate Care Unit, Fondazione IRCCS Cà Granda Ospedale Maggiore Policlinico, 20122 Milano, Italy

**Keywords:** fatty acids, obesity, vitamin D, vitamin D deficiency, DHA, dietary supplements

## Abstract

Obese children are at high risk of developing vitamin D deficiency. Omega-3 polyunsaturated fatty acids and their derivatives might have a beneficial effect on vitamin D status of obese children, due to their anti-inflammatory action, and increasing its absorption. This multicenter, randomized, double-blind controlled study aims to investigate the effect of vitamin D and docosahexaenoic acid (DHA) co-supplementation for six months on vitamin D status, body composition, and metabolic markers of obese children with vitamin D deficiency. A total of 108 children were enrolled and 73 children completed the study: 33 were supplemented with an oral dose of 500 mg of DHA and 1200 IU/day of vitamin D3 and 41 were supplemented with 1200 IU/day of vitamin D3 + wheat germ oil. At the end of the study, more than 50% of the subjects improved their vitamin D status. However, co-supplementation was not more effective than vitamin D plus wheat germ oil. Fat mass percentage was significantly reduced, and body mass index improved in both groups, even if all the subjects were still obese at the end of the study. Children receiving both vitamin D and DHA presented a higher increase of DHA levels that could be relevant to prevent inflammatory-associated complications of obesity, but they had no effect on vitamin D levels.

## 1. Introduction

Obese children are at high risk of developing vitamin D deficiency (VDD) [1], which, in turn, impacts on glucose homeostasis, insulin resistance (IR), and inflammation, thus exacerbating the negative effects of fat accumulation on overall health [2]. The relationship between obesity and low levels of circulating 25-hydroxyvitamin D (25(OH)D) has not been completely elucidated. Adipose tissue represents the major extra skeletal targets of vitamin D. It plays an important role as a vitamin D storage site, due to vitamin D fat-solubility, resulting in a lower bioavailability in the obese subjects [3]. An impaired hydroxylation in adipose tissue and 25(OH)D accumulation in fat may be hypothesized to explain this relationship. Anotherhypothesis considers low serum 25(OH)D, a lipophilic compound, the result of vitamin D dilution in fat mass [4]. Animal and in vitro studies point out that vitamin D modulates adipose tissue biology, by inhibitory or stimulatory effects on adipocyte differentiation depending on cell type and stage of differentiation [5]. Vitamin D exerts its effect also by regulating energy metabolism gene expression, preventing excess body fatness, and limiting the expression of inflammatory molecules [3,6,7].

VDD appears to predispose also to further metabolic disturbances including the metabolic syndrome [3,8,9]. Insulin resistance and cardiovascular diseases, two conditions in which pro-inflammatory cytokines, such as tumor necrosis factor-alpha (TNF-α), are critically involved, are recognized comorbidities in obese subjects. The raised level of TNF-α induces insulin resistance in adipocytes and peripheral tissues by impairing the insulin signaling. A condition of chronic low-grade inflammation secondary to the abnormal production of proinflammatory cytokines, including TNF α, is considered the main mechanism leading to endothelial dysfunction in obesity [10].

It is also known that inflammation is associated with low levels of vitamin D [11]. On the contrary, some fatty acids (especially omega 3) have an anti-inflammatory action and vitamin D and DHA co-supplementation have a favorable effect on the metabolic status of patients with diabetes [12,13]. Omega-3 polyunsaturated fatty acids (n-3 PUFAs) and their derivatives, eicosapentaenoic acid (EPA) and docosahexaenoic acid (DHA), have been shown to exert anti-inflammatory and inflammation-resolving effects, thus they are suggested to be relevant to both the prevention and treatment of human diseases with an inflammatory component [14]. Therefore, we speculated that co-administration of vitamin D and DHA (an omega-3 fatty acid) might improve the vitamin D status more than vitamin D supplementation plus wheat germ oil (a compound rich in omega-6 fatty acids) in obese children.

Hence, the primary aim of this study was to investigate if the co-supplementation of vitamin D and DHA was more effective than vitamin D plus wheat germ oil to improve the vitamin D status of obese children. The secondary aims were to evaluate the effect of vitamin D and DHA co-supplementation on body composition and metabolic markers.

## 2. Methods

### 2.1. Study Design

The study was an investigator-initiated multicenter, randomized, double-blind controlled study. It was conducted from March 2015 to March 2020 at the pediatric outpatient clinic of the Fondazione IRCCS Ca’ Granda Ospedale Maggiore Policlinico Milan and of the ASST Rhodense POT Bollate, Milan, Italy. Inclusion criteria were: (1) Body Mass Index (BMI) higher than 95° centile for age [15]; (2) 6 to 14 years of age at the time of enrollment; (3) VDD (circulating 25-hydroxy vitamin D levels <20 ng/mL); and (4) written informed consent of legal caregivers. Exclusion criteria were the presence of malabsorption, ongoing treatment with corticosteroids or anticonvulsants, metabolic alterations of the calcium–phosphorus balance, and other endocrinologic disorders such as thyroid dysfunction, growth hormone deficiency, and endogenous hypercortisolism.

The study was approved by the Ethical Review Board of Fondazione IRCCS Ca’ Granda Ospedale Maggiore Policlinico, Milano Area 2 (reference number 1 July 2014, 1472/2014). All procedures were performed in accordance with the Declaration of Helsinki. Written informed consent was obtained from the caregiver of all subjects.

### 2.2. Intervention

The participants were randomly assigned by a computer-generated randomization sequence to the following two arms:

- GROUP I (vitamin D + DHA): oral administration of vitamin D3 (1200 IU daily) + 500 mg of DHA for 6 months.
- GROUP II (vitamin D + wheat germ oil): oral administration of vitamin D3 (1200 IU daily) + wheat germ oil capsules for 6 months.

Children were instructed to assume the capsules containing DHA or wheat germ oil and vitamin D during the same mealtime. Throughout the duration of the study, all investigators, participants, outcome assessors, and data analysts were blinded to the assigned treatment. The D + DHA and vitamin D + wheat germ oil capsules were identical, indistinguishable by appearance, color, or flavor. Additionally, all patients were included in the same lifestyle intervention program consisting of advice for a healthy diet and regular physical exercise. Nutritional habits were evaluated by a 24-h recall by a dedicated nutritionist during routine visit, after 3 months from the beginning, and at the end of the study. To increase the compliance monthly calls to the parents were performed.

### 2.3. History, Anthropometric, and Laboratory Parameters

Age, sex, and birth weight were recorded at the enrollment. Furthermore, body height (Harpenden stadiometer), body weight, body composition by bioelectric impedance (Tanita BC 420 MA; Sensor Medics, Milano, Italy), arm circumference, waist circumference, and triceps, biceps, subscapular, and sovrailiac skinfold thickness were measured at enrollment, after 3 months, and at the end of the intervention. The values of BMI and fat mass were transformed into a standard deviation score (SDS).

For the laboratory analysis, a 5 mL blood venous sample in EDTA was collected in fasting conditions at each time point of the study. The following parameters were measured, at the enrollment and at the end of the intervention: glucose, insulin, glycohemoglobin (HbA1c), total cholesterol (TC), high-density lipoprotein (HDL), low-density lipoprotein (LDL), triglycerides (TG), apolipoprotein A (ApoA), apolipoprotein B (ApoB), ApoB/ApoA ratio, parathyroid hormone (PTH), aspartate transaminase (AST), alanine transaminase (ALT), gamma-glutamyl transferase (GGT), calcium (Ca), and phosphorus (P). Homeostasis model assessment (HOMA) index was calculated as glucose values expressed in mg/dL multiplied by the insulin expressed in milliunits/L divided by 405 [16].

FA acids were measured at the enrollment and at the end of the intervention. For the analyses, a few drops of blood were put on Whatman 903 collection cards BHT (Sigma-Aldrich), pre-treated, and stored at temperature of −20 °C. Cards were cut and transferred into vials (one vial for each sample) for methylation as described by Marangoni et al. [17]. Next, 2 mL of KCl solution (Sigma-Aldrich, Steinheim, Germany) and 330 μL hexane (Sigma-Aldrich, Steinheim, Germany) were added. Samples were first vortexed and then centrifuged at 3000 rpm for 10 min. Finally, hexane layer (the upper layer) was collected from each vial and transferred into gas chromatography vial for fatty acids profile evaluation with fast gas-chromatographer Master GC fast (Dani), equipped with a 15-m fused silica capillary column OmegawaxTM 100 (Supelco). The gas chromatography results were analysed using Clarity software (Data Apex). Each fatty acid was evaluated as percentage of the total FA. The following FA were considered for comparisons: palmitic acid (C16:0) and stearic acid (C18:0) of the saturated fatty acids (SFA); 16:1n7; oleic acid (C18:1n9) of the monounsaturated fatty acids (MUFA); alpha-linolenic acid (ALA, C18:3n3), eicosapentaenoic acid (EPA, C20:5n3), and docosahexaenoic acid (DHA, C22:6n3) of the n-3 PUFA; and linoleic (LA, C18:2n6), dihomo-gamma-linolenic acid (DGLA, C20:3n6), and arachidonic acid (AA, C20:4n6) of the n-6 PUFA. The following FA ratios were calculated as proxy of inflammatory state: AA/EPA, AA/DHA, and n6/n3 the PUFA balance marker (EPA+DHA)/total PUFA [18,19]. The related enzymatic activities were estimated by proxy from the following product/substrate ratios: stearoyl-CoA desaturase (SCD), from C18:1n9/C18:0, (16); fatty acid desaturase (FADS2), from DGLA/LA, and FADS1 from AA/DGLA and from EPA/ALA and DHA/EPA. The related pro-inflammatory activities were estimated by proxy from the following product/substrate ratios, ARA/DHA, ARA/LA, e omega3 index, EPA+DHA. For a subsample of patients, tumor necrosis factor alpha (TNF-α), routinely assessed in obese children at our centers, was analyzed at baseline and 6 months from plasma, with the Quantikine HS Human TNF-alpha Immunoassay (R&D Systems, Minneapolis, MO, USA).

Circulating levels of 25(OH)D were measured by an Abbott chemiluminescent microparticle immunoassay. Reliability and accuracy of the assay were assessed both in the Vitamin D External Quality Assessment Scheme and in the Vitamin D Standardization Program [20,21]. All analyses were performed at the central laboratory of the Fondazione IRCCS Ca’ Granda Ospedale Maggiore Policlinico, Milano, Italy.

### 2.4. Statistical Analysis

Assuming an increase of vitamin D levels of 40% and 55% in the group receiving vitamin D plus wheat germ oil and in the group receiving vitamin D plus DHA, respectively, and the possibility of some dropouts (approximately 10%), a sample size of 108 children is necessary to achieve a power of 80% with a 95% significance level. Data distribution was checked for normality by the Kolmogorov–Smirnov test. Continuous variables were expressed as mean ± SD or as median and interquartile range (IQR). Categorical data were expressed as absolute and relative frequency. Data were analyzed using the per-protocol principle and the values recorded at baseline were compared to values recorded at 6 months. Baseline and follow-up characteristics were tested for differences by Student’s t-test or Mann–Whitney test, as appropriate. The change of anthropometric and laboratory values between baseline and 6 months was evaluated using the Wilcoxon test for repeated measures. Difference between proportions were tested using the chi-squared test. Delta differences (∂) between the start and the end of treatment were calculated as ∂ = [(T1−T0)/T0] × 100. Univariate correlations were investigated with Spearman’s rank correlation test. Logistic regression analysis was used to test the independence of associations between vitamin D levels (≥20 ng/mL vs. <20 ng/mL) at the end of the study and the interventional group, DHA levels, and anthropometric variables. Missing data were handled using complete case analysis. Significance was assumed for *p* < 0.05. The data were analyzed using SPSS (Statistical Package for Social Science v.20 Inc., Chicago, IL, USA).

## 3. Results

### 3.1. Baseline Characteristics

**Figure 1:**
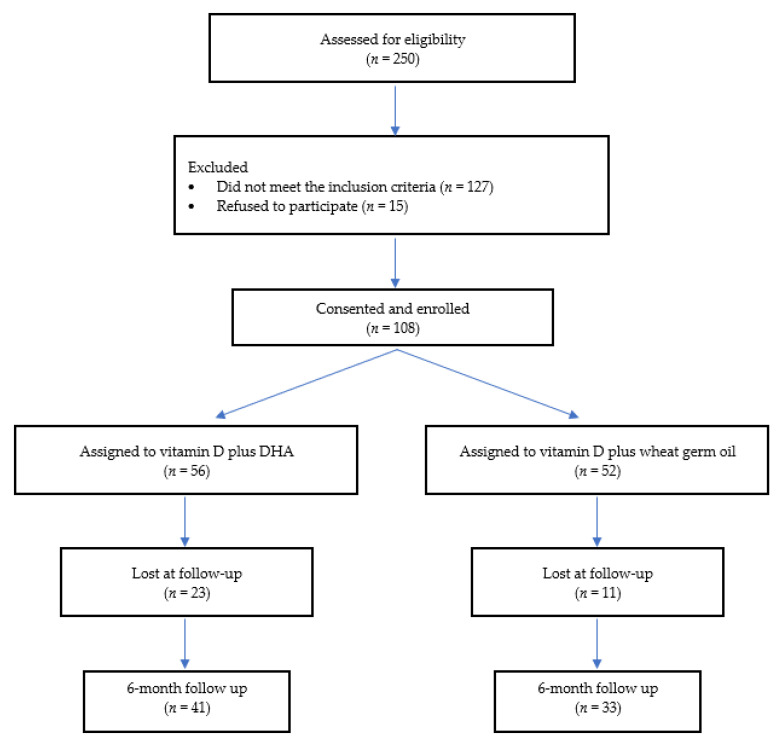
Enrollment flow chart.

A total of 250 patients were screened in the two study centers and 108 were finally enrolled, as reported in Figure 1. Of these, 74 (45 males, 29 females), (median age 11.2 (IQR 3.2) years) completed the study. A total of 34 patients were lost at follow-up (a comparison of the main baseline characteristics of subjects who completed the study and dropouts are reported in the supplementary online materials). The dropouts were due to the refusal of parents or their children to continue the supplementation. Among subjects who completed the study, 33 were in the GROUP I and 41 children were in GROUP II. The two groups showed similar baseline characteristics, as shown in Table 1.

At baseline, median values (IQR) of vitamin D were 14.2 (7.8) ng/mL. In both groups, the concentration of vitamin D increased during the study period. At the end of the 6 months, in GROUP I, median vitamin D levels were 21.9. (IQR 11.8) ng/mL and in GROUP II were 23.4 (8.8) ng/mL, thusresulting in a median (IQR) increase by 64.3% and 70% in the two groups, respectively. Only for 4 patients vitamin D concentrations decreased (3 patients in GROUP I, 1 in GROUP II). However, at the end of the study, 28 (38%) patients (16 patients in vitamin GROUP I and 12 in GROUP II) had persistent VDD (median vitamin D 15.7 (IQR 4.1) ng/mL).

Logistic regression analysis showed that vitamin VDD at the end of the study was independently associated with the percentage of fat (OR = 1.10, 95% CI = 1.01–1.21, *p*-value = 0.037) (Table 2). No association was found between VDD and the assigned interventional group.

### 3.2. Anthropometric, Clinical, and Laboratory Parameters

Table 3 shows the anthropometric and laboratory characteristics for each group at baseline and at the end of intervention The two groups showed a reduction in SDS BMI % (−2.9 in GROUP I, −7.1 in GROUP II). There was a major reduction of fat mass in GROUP II (−3.5%) than GROUP I (0.62%). GROUP II showed a reduction in median (IQR) levels of ApoA (from 138.5 (25) mg/dL at baseline to 132(29) mg/dL at 6-months *p* = 0.018; ∂ −6.2%). The ApoB/ApoA ratio was significantly increased in both groups (∂: +8.4% in GROUP I and +3.5% in GROUP II, *p* = 0.030). No other significant differences for any anthropometric and laboratory parameters were found at 6 months.

### 3.3. Fatty Acids

Table 4 shows the fatty acids concentration at baseline and at the end of the study in the two groups. Median (IQR) DHA % was higher in GROUP I (2.56 (1.15)); thus resulting in an increase of 58% vs. an increase of 35% in GROUP II (*p* = 0.010). Differences were found also for total n3-PUFA, PUFA balance, AA/DHA, n3 derivatives/ALA, DHA/EPA, DHA/ALA, and EPA+DHA, as shown in Table 4. The concentration of DHA and total n3-PUFA increased in both groups. Consequently, PUFA balance and the following ratios (n3-derivatives/ALA, DHA/EPA, and DHA/ALA) increased, too. In GROUP I, 16:1n7, SCD16, total MUFA, AA/EPA, AA/DHA and n6/n3 decreased. In GROUP II, the concentration of 16:0, AA/DHA, AA/EPA, n6/n3 decreased. No significant correlations of serum 25(OH)D concentrations with 20:4n6; 22:6n3; 20:5 n3 were found either at baseline or at 6 months in both groups (data not shown).

### 3.4. TNF-α

In 28 patients (14 per group) TNF-α was measured. The median TNF-α value was 6.6 (IQR 9.6) pg/mL at baseline and 5.2 (7.6) pg/mL at 6 months. In GROUP I, the median TNF-α value was 5.5 (IQR 7.9) pg/mL, and 6.1 (7.9) pg/mL, at baseline and at 6 months, respectively, and in GROUP II the median TNF-α value was 7.2 (IQR 9.7) pg/mL and 4.6 (7.2) pg/mL at baseline and at 6 months, respectively. There was a significative reduction in TNF-α levels between the two groups (*p* = 0.048). 

## 4. Discussion

This study aimed to investigate the potential benefit of supplementing VDD obese children with vitamin D3 plus DHA as compared to vitamin D supplementation plus wheat germ oil. We hypothesized that the association of DHA with vitamin D supplements could help improve the vitamin D status, body composition, and metabolic markers of this population. Regarding the primary outcome—vitamin D blood levels—more that 50% of the subjects improved their vitamin D status, regardless of the supplementation assigned. Children with persistent VDD at the end of the study were about 38% of the sample. This result could be explained by different reasons. Firstly, in our study vitamin D supplementation was daily based, which may have represented a challenge to some patients in terms of compliance. Secondly, we did not investigate the sunexposure and the season on which the supplementation was provided [22]. Lastly, the dose of vitamin D might be too low to obtain significant results: previous studies reported an increase of serum 25(OH)D after supplementation of 1000–2000 IU/day but not after 600 IU/day in overweight/obese children [23,24]. Castaneda and colleagues [25] found a significantly lower vitamin D increase in obese adolescents compared to non-obese subjects, after supplementation of 2000 IU/day, thus confirming that a higher vitamin D dose in obese subjects is needed to treat VDD.

The secondary aim of the study was to test the effects of vitamin D plus DHA DHA on BMI and body composition. The nutritional outcome was ameliorated, but not significantly changed in terms of BMI among the included subjects, who were still in condition of obesity at the end of the study. This finding is likely due to the length of the study that may not be sufficient to detect significant differences in subjects with obesity [26]. Nevertheless, the nutritional outcome was overall ameliorated in terms of the FM% which was significantly reduced in both groups. This is probably the result of the dietary and lifestyle counseling provided to all subjects at the beginning of study, according to international and national guidelines on nutritional management of obesity in pediatric subjects. As expected, we found that FM% at the end of the study was independently associated with VDD status of the included subjects. Previous studies showed similar results in term of BMI [27], while others suggested a dose-related effect on BMI, waist circumference, and total fat mass [28].

The present study also investigated the fatty acids profile in both groups. To the best of our knowledge, little is known about the effect of PUFA supplementation in obese children. Supplementation with DHA in children with non-alcoholic fatty liver disease showed a decrease of fatty liver [28] and a significant reduction in pericardiac and visceral fat, and also in triglycerides and fasting insulin after 6 months of treatment [29]. As expected, in the group supplemented also with DHA, this n-3 fatty acid was increased after 6 months compared with the subjects supplemented with vitamin D plus wheat germ oil. Specifically, DHA were increased by more than 50%, in the group receiving DHA and only by 35% in the other group. Similar findings were reported by López-Alarcón [30] showing a significantly increased EPA and DHA after 3 months of LC-PUFA supplementation. The increase of DHA also in the group receiving wheat germ oil might be explained by the improvement of dietary habits, such as increased consumption of fish.

In the past decade, evidence suggested that obesity induces low-grade chronic inflammation, affecting the immune and metabolic state [31]. Moreover, markers of oxidative stress and inflammation are shown to be elevated in people with low serum 25(OH)D concentrations; however, results are not always consistent [32,33,34,35]. In our study TNF-α levels were found to be slightly, but still significantly, decreased in both groups. A recent study [36] investigated whether dietary fat and/or antioxidant intake influences circulating TNF-α, interleukin-6 (IL-6), CRP, and leptin concentrations. There was a significant increase in CRP, IL-6, and leptin, but not in TNF-α, with increasing adiposity, independent of age. Another study showed that inflammatory markers are increased in overweight children as young as 6 years old [37]. Moreover, there is growing evidence about the role of total fat intake and specific fatty intake on systemic inflammation: C18:2 might increase IL-6 and other inflammatory cytokines production in the adipose tissue, whereas α-linolenic acid might reduce inflammation [38]. However, we speculate that the decrease of TNF-α in both the studied groups might be associated both to the BMI decrease and the dietary changes.

Previous studies found that n-3 FA treatment was able to attenuate metabolic disorders associated with obesity, by limiting HF-induced glucose intolerance and liver steatosis, and mice fed n-3 FA displayed lower circulating leptin [39]. On the contrary, vitamin D3 supplementation did not enhance the benefits observed with n-3 FA on plasma leptin levels, glucose tolerance, the liver lipid metabolism, or intestinal health. Moreover, the coadministration of n-3 FA and D3 significantly reduced the increase in circulating 25(OH)D following D3 consumption. Some authors hypothesized that n-3 FA–based bile acid micelles have an increased size that would compromise the diffusion of micelles containing D3. They also suggested that n-3 FA supplements may impede the efficacy of D3 supplementation in obesity. Yet, these assumptions warrant further mechanistic investigations and validation studies [39].

A few limitations deserve to be commented. This study compared two active groups without including a placebo group, because our study included children with a deficit of vitamin D who must receive vitamin D according to Italian guidelines [40]. Therefore, it would have been unethical to include a placebo group. Furthermore, the interventional period was limited.

## 5. Conclusions

In conclusion, this study shows that vitamin D and DHA co-supplementation for 6 months does not lead to a better vitamin D status as compared to vitamin D plus wheat germ oil. We observed a beneficial effect on the DHA levels in children supplemented with this FA. It might be speculated that the anti-inflammatory action of n-3 PUFA may in part compensate for the detrimental outcome of VDD. Understanding the relationship among obesity, vitamin D status, and fatty acids profile is clinically relevant, and the investigation of the underlying pathogenic mechanisms may expand clinical approach to obesity.

## Figures and Tables

**Table 1:** Clinical and anthropometric variables in D + DHA group and vitamin D group after 6 months from the start of the study. Data are expressed as median (IQR).

	Group	Baseline	6 Months
Sex (M/F)	45/29		
Age, years	I	11.9 (3.1)	12.0 (3.1)
	II	11.0 (3.5)	11.6 (3.1)
Gestational Age, weeks	I	37.0 (3.0)	
	II	37.0 (3.0)	
Birth weight, g	I	3250 (767.5)	
	II	3300.0 (730.0)	
Age at obesity diagnosis, years	I	7 (2.0)	
	II	6.5 (3.8)	
Body height, cm	I	150.0 (15.8)	154.0 (15.0) ***
	II	145.0 (15.3)	149.2 (15.0) ***
Body weight, kg	I	61.5 (24.0)	64.7 (23.9) *
	II	56.0 (19.4)	58.5 (20.4) **
SDS BMI	I	2.53 (0.64)	2.5 (0.6)
	II	2.63 (0.70)	2.5 (0.7) **
AM, cm	I	28.3 (3.9)	28.5 (5.0)
	II	27.0 (3.8)	28.0 (4.0)
WC, cm	I	84.3 (16.6)	84.0 (17.0)
	II	81.5 (12.4)	83.0 (10.0)
Biceps skinfold, mm	I	19.8 (8.0)	19.0 (5.3)
	II	19.5 (6.8)	20.0 (6.6)
Triceps skinfold, mm	I	26.0 (7.1)	26.0 (4.6)
	II	24.3 (7.8)	24.0 (4.5)
Sovrailiac skinfold, mm	I	26.0 (7.0)	25.0 (7.5)
	II	24.2 (7.6)	25.0 (8.0)
Subscapular skinfold, mm	I	23.6 (10.7)	21.0 (12.0)
	II	23.3 (6.4)	23.0 (9.0)
Fat Mass, %	I	35.0 (8.3)	35.4 (8.4)
	II	35.3 (5.5)	33.4 (7.5)
Fat Mass SDS, %	I	5.87 (2.45)	1.42 (0.85) ***
	II	6.38 (3.05)	1.36 (0.58) ***
FFM, kg	I	37.7 (11.6)	41.4 (12.8) ***
	II	35.1 (13.2)	39.1 (12.2) ***

Baseline vs. 6 months (Wilcoxon test): * *p* < 0.05; ** *p* < 0.01; *** *p* < 0.001. SDS = Standard Deviation; AM = Arm Circumference; FFM = Fat Free Mass; WC =Waist Circumference.

**Table 2:** Logistic regression showing the association between VDD (dependent variable) and interventional arm, percentage of fat, BMI status and DHA levels at the end of the study.

	OR	95% CI		*p*-Value
Follow-Up				
22:6n3	1.65	0.85	3.21	0.138
Fat %	1.11	1.01	1.21	0.037
BMI SDS	0.78	0.22	2.70	0.694
Group II	1.42	0.46	4.40	0.548

22:6n3 = Docosahexaenoic Acid (DHA); BMI = Body Mass Index; SDS = Standard Deviation.

**Table 3:** Laboratory/biochemical variables in D + DHA group and vitamin D group at baseline and after 6 months. Data are expressed as median (IQR).

	Group	Baseline	6 Months
Glucose, mg/dL	I	85.0 (12.0)	87.0 (10.0)
	II	86.0 (5.0)	85.0 (8.8)
Insulin, mg/dL	I	15.5 (17.5)	17.8 (15.6)
	II	15.7 (9.7)	15.4 (12.0)
HOMA	I	3.0 (4.0)	3.5 (3.3)
	II	3.1 (2.4)	3.2 (2.5)
HbA1c (%)	I	5.3 (0.4)	5.2 (0.4)
	II	5.3 (0.3)	5.3 (0.3)
TC (mg/dL)	I	153.5 (40.8)	154.0 (32.0)
	II	158.0 (31.0)	158.0 (36.5)
HDL-c (mg/dL)	I	51.0 (17.3)	49.0 (17.0)
	II	50.0 (16.0)	49.0 (17.8)
TG (mg/dL)	I	78.5 (60.8)	66.0 (33.0)
	II	69.0 (59.0)	73.5 (57.8)
LDL-c (mg/dL)	I	84.0 (28.0)	83.0 (33.0)
	II	88.0 (35.1)	89.0 (36.7)
ALT (UI/L)	I	21.5 (8.5)	23.5 (10.0)
	II	19.0 (15.5)	26.0 (17.0)
AST (UI/L)	I	23.0 (7.5)	26.0 (6.8)
	II	25.0 (7.5)	28.0 (10.0)
GGT (UI/L)	I	13.0 (8.0)	15.5 (10.8)
	II	13.0 (8.5)	14.5 (7.5)
ApoA (mg/dL)	I	138.5 (25.0)	132.0 (29.0) *
	II	137.0 (25.5)	130.0 (26.3)
ApoB (mg/dL)	I	81.0 (24.5)	79.0 (30.0)
	II	77.0 (26.5)	81.5 (24.5)
B/A	I	0.59 (0.19)	0.58 (0.19) *
	II	0.55 (0.15)	0.57 (0.23) *
PTH (pg/mL)	I	28.1 (27.0)	33.6 (24.8)
	II	36.5 (25.5)	31.8 (24.9)
Calcium (mg/dL)	I	9.76 (0.40)	9.71 (0.37)
	II	9.74 (0.50)	9.73 (0.54)
25OHD (ng/mL)	I	14.0 (7.2)	21.99 (11.8)
	II	15.3 (8.4)	23.4 (8.8) ***

Baseline vs. 6 months (Wilcoxon test): * *p* < 0.05; *** *p* < 0.001. HOMA = Homeostasis Model Assessment; HbA1c = Glycohemoglobin; TC = Total Cholesterol; HDL-c = High-Density Lipoprotein Cholesterol; TG = Triglycerides; LDL-c = Low-Density Lipoprotein Cholesterol; ASL = Aspartate Transaminase; ALT = Alanine Transaminase; GGT = ɣ-glutamyl-transferase; ApoA = Apolipoprotein A; ApoB = Apolipoprotein B; B/A = ApoB/ApoA Ratio; PTH = Parathyroid Hormone; 25OHD = 25-hydroxy Vitamin D.

**Table 4:** Percentage of fatty acids distribution in vitamin D + DHA subjects (Group I) and vitamin D + wheat germ oil subjects (GROUP II) at baseline and after 6 months. Data are expressed as median (IQR).

% of Total Fatty Acids	Baseline		6 Months	
	GROUP I	GROUP II	GROUP I	GROUP II
16:00	23.65 (1.89)	23.61 (1.98)	23.53 (1.75)	23.23 (1.64) *
18:00	11.90 (2.10)	11.74 (1.75)	11.58 (2.30)	11.43 (2.19)
16:1n7	1.42 (0.67)	1.41 (0.69)	1.18 (0.59) *	1.22 (0.81)
18:1n9	18.36 (3.43)	18.60 (3.14)	18.30 (2.56)	17.96 (2.93)
18:1n7	1.30 (0.26)	1.30 (0.27)	1.31 (0.38)	1.31 (0.22)
18:2n6	21.07 (3.24)	21.31 (2.88)	21.34 (3.36)	20.86 (3.80)
20:3n6	1.60 (0.40)	1.59 (0.29)	1.56 (0.31)	1.57 (0.45)
20:4n6	9.41 (3.05)	9.61 (2.24)	9.38 (1.99)	9.89 (1.77)
22:4n6	1.20 (0.56)	1.19 (0.56)	1.02 (0.45)	1.23 (0.49)
18:3n3	0.19 (0.08)	0.19 (0.08)	0.17 (0.08)	0.20 (0.09)
20:5n3	0.30 (0.27)	0.23 (0.19)	0.26 (0.17)	0.29 (0.20)
22:5n3	0.54 (0.24)	0.58 (0.31)	0.47 (0.26)	0.57 (0.20)
22:6n3	1.64 (0.81)	1.65 (0.71)	2.59 (1.15) ***	2.06 (1.35) *** °
Total SFA	39.68 (1.96)	38.56 (3.92)	38.73 (4.53)	38.21 (3.34)
Total MUFA	23.35 (3.20)	23.21 (3.45)	22.97 (2.47) *	22.78 (3.44)
Total PUFA	36.90 (4.16)	37.61 (6.38)	37.49 (6.19)	37.80
Total n6-PUFA	34.14 (3.80)	35.10 (5.69)	34.19 (5.18)	34.81 (5.27)
Total n3-PUFA	2.84 (1.02)	2.67 (0.88)	3.71 (0.99) ***	3.14 (1.68) *** °
LCPUFA	14.97 (4.59)	15.57 (2.40)	15.91 (2.34)*	15.73 (3.19)
PUFA balance	5.50 (2.27)	5.25 (2.00)	7.87 (2.71) ***	5.87 (4.01) *** °
AA/EPA	29.91 (27.15)	35.56 (22.59)	4.16 (25.94) ***	7.78 (23.89) ***
AA/DHA	5.43 (2.73)	5.76 (2.09)	3.40 (1.72) ***	4.75 (4.75) *** °
n6/n3	21.85 (30.05)	21.05 (33.44)	9.35 (3.00) ***	11.00 (4.95) ***
n6-derivates/LA	1.59 (0.22)	1.62 (0.13)	1.60 (0.16)	1.65 (0.15)
n3-derivates/ALA	15.05 (6.49)	14.70 (6.56)	22.37 (12.03) ***	17.51 (9.33) °
DGLA/LA (FADS2)	0.07 (0.03)	0.07 (0.03)	0.07 (0.02)	0.08 (0.03)
ARA/DGLA (FADS1)	5.64 (1.54)	5.89 (1.79)	5.89 (1.42)	6.11 (2.18)
ARA/LA	0.42 (0.16)	0.45 (0.09)	0.44 (0.13)	0.48 (0.13)
EPA/ALA	1.53 (1.38)	1.39 (1.12)	1.65 (1.14) ***	1.44 (1.00)
DHA/EPA	5.55 (4.48)	5.63 (4.50)	10.22 (8.17) ***	7.69 (4.16) *** °
DHA/ALA	9.11 (4.23)	9.33 (4.44)	17.27 (10.69) ***	12.03 (6.68) *** °
SCD(16)	0.06 (0.03)	0.06 (0.03)	0.05 (0.02) *	0.05 (0.03)
SCD(18)	1.55 (0.42)	1.63 (0.39)	1.55 (0.42)	1.55 (0.37)
EPA+DHA	2.01 (0.97)	1.88 (0.83)	2.84 (1.17) ***	2.28 (1.41) *** °

Baseline vs. 6 months (Wilcoxon test): * *p* < 0.05; *** *p* < 0.001. D + DHA group vs. vitamin D at baseline and 6 months (Mann–Whitney test): ° *p* < 0.05. 16:00 = Palmitic Acid; 18:0 Stearic Acid; 16:1n-7 = Palmitoleic Acid; 18:1n7 = Vaccenic Acid; 18:1n-9 = Oleic Acid; 18:2n6 = Linoleic Acid; 20:3n6 = Dihomo-γ-linolenic Acid; 20:4n6 = Arachidonic Acid; 22:4n6 = Adrenic acid; 18:3n3 = Alpha-linoleic Acid; 20:5n3 = Eicosapentaenoic Acid; 22:5n3 = Docosopentaenoic Acid; 22:6n3 = Docosahexaenoic Acid; SFA = Saturated Fatty Acids; MUFA = Monounsaturated Fatty Acids; PUFA = Polyunsaturated Fatty Acids; LCPUFA = Long Chain Polyunsaturated Fatty Acids; AA = Arachidonic Acid; EPA = Eicosapentaenoic Acid; DHA = Docosahexaenoic Acid; DGLA = Dihomo-γ-linolenic Acid; LA = Linoleic Acid; FADS = Fatty Acid Desaturase; ALA = Alpha-linoleic Acid; SCD = Stearoyl-CoA Desaturase.

## Data Availability

Data are available upon reasonable request from the corresponding author.

## Supplementary Materials

The following supporting information can be downloaded at: https://www.mdpi.com/article/10.3390/nu14071397/s1, Supplementary Table S1: Median (IQR) clinical and anthropometric variables in the total groups of patients who completed the study and in the drop-outs group at baseline; Supplementary Table S2: Median (IQR) clinical and anthropometric variables of patients who completed the study and of the drop-outs patients in vitamin D +DHA group at baseline; Supplementary Table S3: Median (IQR) clinical and anthropometric variables of patients who completed the study and of the drop-outs patients in vitamin D group at baseline.
