# Supplementary material for: Effect of Vitamin D and Docosahexaenoic Acid Co-Supplementation on Vitamin D Status, Body Composition, and Metabolic Markers in Obese Children: A Randomized, Double Blind, Controlled Study
Source: Nutrients. 2022 Mar 27;14(7):1397. doi: 10.3390/nu14071397 (PMC9003047; doi:10.3390/nu14071397)
Supplement: Supplementary file 1 [file nutrients-14-01397-s001.zip › nutrients-1632868-supplementary.pdf]

nutrients

MDPI

Supplementary Materials

Effect of Vitamin D and Docosahexaenoic Acid Co-Supplementation on Vitamin D Status, Body Composition, and Metabolic Markers in Obese Children: A Randomized, Double Blind, Controlled Study

Valentina De Cosmi [1], Alessandra Mazzocchi [1], Veronica D'Oria [2], Alessandro Re [3], Giulia Carla Immacolata Spolidoro [1], Gregorio P. Milani [1,4,*], Cristiana Berti [4], Silvia Scaglioni [5], Claudia Giavoli [1,6], Silvia Bergamaschi [6], Giulia Rodari [6], Eriselda Profka [6], Roberto Colombo [3] and Carlo Agostoni [1,7]

Table S1. Median (IQR) clinical and anthropometric variables in the total groups of patients who completed the study and in the drop-outs group at baseline.

Total population	Patients who completed the study		Drop-out		*p*-value
	Median	IQR	Median	IQR	
Age, years	11.2	3.2	8.7	3.8	0.000*
SDS BMI	2.6	0.6	2.6	0.6	0.883
FM, %	35.3	7.4	36.4	7.5	0.553
FFM, kg	36.9	10.9	37.9	13.6	0.901
25OHD	14.4	8.0	15.2	5.6	0.499

**Mann-Whitney test $p < 0.05$.* IQR = interquartile range; FM = fat mass; FFM = fat free mass; SDS = standard deviation score; 25OHD = 25-hydroxy vitamin D.

Table S2. Median (IQR) clinical and anthropometric variables of patients who completed the study and of the drop-outs patients in vitamin D +DHA group at baseline.

Vitamin D + DHA group	Patients who completed the study		Drop-out		*p*-value
	Median	IQR	Median	IQR	
Age, years	11.1	2.5	8.7	4.1	0.007*
SDS BMI	2.6	0.6	2.6	0.8	0.752
FM, %	34.4	6.4	36.0	8.7	0.383
FFM, kg	37.3	11.0	35.7	13.3	0.605
25OHD	14.0	6.8	15.0	6.0	0.542

**Mann-Whitney test $p < 0.05$.* IQR = interquartile range; FM = fat mass; FFM = fat free mass; SDS = standard deviation score; 25OHD = 25-hydroxy vitamin D.

Table S3. Median (IQR) clinical and anthropometric variables of patients who completed the study and of the drop-outs patients in vitamin D group at baseline.

Vitamin D group	Patients who completed the study		Drop-out		*p*-value
	**Median**	**IQR**	**Median**	**IQR**	
Age, years	11.6	3.7	9.0	4.2	0.021*
SDS BMI	2.6	0.7	2.6	0.7	0.968
FM, %	21.0	10.5	23.1	14.8	0.873
FFM, kg	36.5	11.1	40.2	19.7	0.766
25OHD	15.3	8.4	15.7	4.1	0.582

**Mann-Whitney test* $p<0.05$. IQR = interquartile range; FM = fat mass; FFM = fat free mass; SDS = standard deviation score; 25OHD = 25-hydroxy vitamin D.

We found that the only differences between drop-outs and the patients who completed the study (in the total group, and in each group) was related to age, but it was not clinically relevant.
